# Feasibility of Using Clinical Element Models (CEM) to Standardize Phenotype Variables in the Database of Genotypes and Phenotypes (dbGaP)

**DOI:** 10.1371/journal.pone.0076384

**Published:** 2013-09-18

**Authors:** Ko-Wei Lin, Melissa Tharp, Mike Conway, Alexander Hsieh, Mindy Ross, Jihoon Kim, Hyeon-Eui Kim

**Affiliations:** Division of Biomedical Informatics, Department of Medicine, School of Medicine, University of California San Diego, La Jolla, California, United States of America; CSIR-Institute of Microbial Technology, India

## Abstract

The database of Genotypes and Phenotypes (dbGaP) contains various types of data generated from genome-wide association studies (GWAS). These data can be used to facilitate novel scientific discoveries and to reduce cost and time for exploratory research. However, idiosyncrasies and inconsistencies in phenotype variable names are a major barrier to reusing these data. We addressed these challenges in standardizing phenotype variables by formalizing their descriptions using Clinical Element Models (CEM). Designed to represent clinical data, CEMs were highly expressive and thus were able to represent a majority (77.5%) of the 215 phenotype variable descriptions. However, their high expressivity also made it difficult to directly apply them to research data such as phenotype variables in dbGaP. Our study suggested that simplification of the template models makes it more straightforward to formally represent the key semantics of phenotype variables.

## Introduction

With the advancements in genome-wide association studies (GWAS), the number of public genotypic and phenotypic data repositories, such as the database of Genotypes and Phenotypes (dbGaP), has significantly increased [1,2]. The use or reuse of GWAS data can promote exploratory research, validate existing findings, and reduce research time and costs. However, data in public repositories are not always collected in a standardized or harmonized way, making it difficult to reuse these data. A Phenotype, as defined and reported in GWAS studies, is a characteristic or trait of interest, which is any observation ranging from disease risk to physical properties (e.g., blood pressure, disease onset age, disease condition, premature days, height). Standardization of phenotype data is particularly challenging.

As shown in Table **1**, phenotype variables are often named without a specific naming convention, or are often labeled with abbreviated codes that do not convey clear meaning. Many of these variables are accompanied by descriptions that help users understand what data the variable intends to represent. However, keyword searches applied to variable descriptions do not always provide accurate results due to syntactic and lexical complexities associated with the descriptions such as use of negation and synonyms [3].

**Table 1:** Idiosyncratic height variable representation in dbGaP.

**Variable ID**	**Variable Names**	**Variable Descriptions**
phv00071000.v1	Htcm	Standing height at follow up visit
phv00165340.v1.p2	ESP_HEIGHT_BASELINE	Standing height in cm at baseline
phv00083471.v1.p2	lunghta4	HEIGHT (cm)

Idiosyncrasies in variable names play a major hurdle to utilizing the data stored in dbGaP and are the focus of this paper. As a first step towards standardizing the phenotype variables in dbGaP, we tested the adequacy of an existing information model for clinical data, the Clinical Element Models (CEM), developed by GE Healthcare/Intermountain Healthcare Data Modeling and Terminology Team [4] to formally represent phenotype variable descriptions in dbGaP. Our intention was to test the feasibility of using the CEMs as a type system for the natural language processing (NLP) algorithms that standardize phenotype variables in dbGaP by identifying key semantics and representing them using a formal structure.

For our feasibility testing, we evaluated (1) the content coverage of existing CEMs on a small set of phenotype variables, and (2) the feasibility of formalizing phenotype variable descriptions using CEM template models.

## Background

### Challenges in standardizing phenotype variables in dbGaP

dbGaP contains various types of data generated in many GWAS studies, such as phenotypes, genotypes, and pedigree information of subjects, as well as specifics on samples, measurements and experiments. As of July 2013, dbGaP contains more than 420 studies, which in turn hold more than 2,600 data sets and 137,000 variables [2]. Although dbGaP contains abundant phenotype variables and provides a web-based user interface for searching studies by phenotypes of interest, idiosyncrasies in the variable names make it difficult to identify relevant studies with a sufficient level of accuracy [5].

The Phenotype Finder IN Data Resources (PFINDR) initiative, put forth by the National Heart, Lung, and Blood Institute (NHLBI), aims to make various phenotype data available for GWAS related investigations. Challenges associated with non-standardized phenotype variables generated in different research institutions are widely recognized [6]. The eMERGE (Electronic medical Records and Genomics) Network [7], funded by the National Human Genome Research Institute (NHGRI), is another project dealing with the use of phenotypes collected in the electronic medical record to support GWAS. Standardization of the phenotype variables collected from different institutions/studies is a common challenge for these initiatives [7].

eMERGE aims to make clinical data in electronic health record (EHR) available for GWAS. In eMERGE, phenotype variables are standardized through detailed semantic annotation, including mapping to standardized terminology systems and data elements [8,9]. In eMERGE, the phenotype data are standardized during the submission process through metadata annotation and mapping to existing standards such as National Cancer Institute Thesaurus (NCIT) [10], cancer Data Standard Registry and Repository (caDSR) [11], Study Data Tabulation Model (SDTM) [12], and Systematized Nomenclature of Medicine-Clinical Terms (SNOMED-CT) [13]. Users can search and browse through standardized phenotype variables and their metadata using eleMAP, a web-based tool for managing phenotype variables developed by eMERGE [7]. Users from the participating institutions register their data to eleMAP in a standardized format using a provided template [9]. Similarly, we plan to adopt semantic annotation as part of the standardization of phenotype variables in Phenotype Discoverer (PhenDisco), a project funded through PFINDR.

Both the PFINDR program and the eMERGE network aim to standardize phenotype variables. However, unlike eMERGE, PFINDR deals with the large amount of phenotype data already stored in dbGaP lacking representational standards. Manually standardizing such a huge set of data would be cumbersome and prohibitively costly. Therefore, devising an algorithmic means of processing the existing phenotype variables in dbGaP is crucial to this task. As a first step, we needed to develop a systematic method of identifying core semantics from the variable descriptions.

The Strategic Health IT Advanced Research Projects (SHARPn) are closely related to eMERGE activities. The SHARPn were instigated by the Office of the National Coordinator for Health Information Technology to address key obstacles to the adoption of Electronic Health Records (EHR) such as security of health information and building shared network architectures. In particular, its fourth project [14,15,16], focuses on the secondary use of Electronic Health Records to improve healthcare. A vital task in facilitating secondary use is the development of appropriate clinical models and NLP tools to convert the information currently encoded in EHR free-text fields to structured data.

Natural Language Processing (NLP) has proven effective in determining semantic categories and relations in the biomedical domain. For example, the Genomics Information Extraction System (GENIES) extended an existing NLP system to identify categories, a lexicon, and a grammar [17]. Navigli and colleagues reported extracting both taxonomic and non-taxonomic relations between concepts based on existing domain ontologies [18]. In another project, SemSpec utilizes an existing NLP system to extract hypernymic propositions, through syntactic structures in the text and knowledge from a domain ontology [19]. The success of these systems shows that NLP can help in determining semantic categories and relations in biomedical text that carry core information delivered in the text. In these studies, formal representations of text, serving as a model system for NLP, played a crucial role.

### Existing information and terminology models

**Figure 1:**
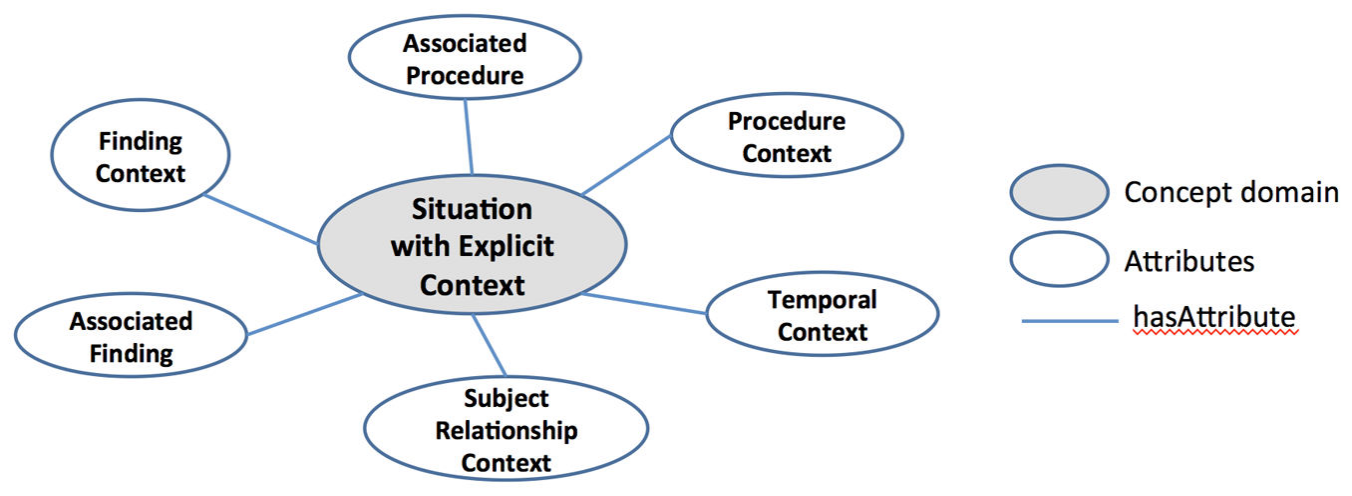
Attributes used to define *Situation with Explicit* Context concepts in the Systematized Nomenclature of Medicine, Clinical Terms (SNOMED-CT). SNOMED-CT system provides concept models constructed in different clinical domains. An example of a concept model for *Situation* *with* *Explicit* *Context* is presented. This model includes six attributes: Associated Procedure, procedure Context, Temporal Context, Subject relationship Context, Associated finding, and Finding Context. Grey oval: concept domain; white oval: attributes; blue line: hasAttribute.

Existing terminology and information model standards provide conceptual models for formally representing a healthcare domain. For example, the Systematized Nomenclature of Medicine, Clinical Terms (SNOMED-CT) system provides the concept models, from which a concept is constructed, in 9 different clinical domains such as *Clinical Findings, Procedures, Evaluation Procedures, Specimen, Body Structure, Pharmaceutical/Biological Product, Situation with Explicit Context, Event*, and *Physical Object* [20]. As an example, the concept model for *Situation with Explicit Context* is presented in Figure **1**. SNOMED-CT is a compositional terminology and these conceptual structures primarily serve as the syntaxes for concept composition [21].

**Figure 2:**
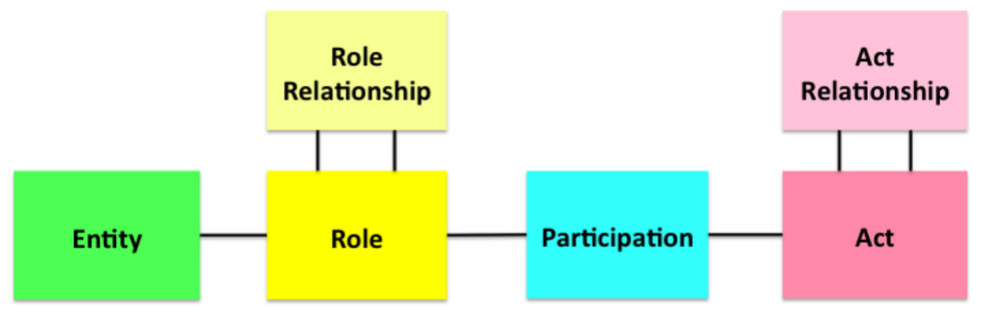
Reference Information Model (RIM) core domain. RIM of Health Level 7 (HL7), an existing terminology and information model standards, uses an object-oriented modeling approach derived from four main classes: Act, Entity, Role, and Participation (https://wiki.nci.nih.gov/display/SAIF/HL7+Reference+Information+Model).

The Reference Information Model (RIM) of the Health Level 7 (HL7) is an example of an information model standard [22]. RIM provides a shared view on the healthcare domain, from which a message is generated, regardless of the message structure [22] (Figure **2**). RIM describes the healthcare domain using an object-oriented modeling approach based on 4 major constructs such as *Act*, *Entity*, *Role*, and *Participation*, each of which is further described using various classes and their associated attributes.

Although both SNOMED-CT concept models and RIM provide a means of formally describing any given healthcare domain, their limited scope and approach to representation make them less plausible options for representing phenotype variables in dbGaP. The SNOMED-CT concept models provide sophisticated ways of expressing a complex clinical concept in a certain clinical domain by attaching multiple attribute concepts (i.e., qualifiers and modifiers) to a key concept. While it offers a very high level of sophistication for representing a unit of clinical concept, it lacks recursive or nested structures, which are often required for representing a phenotype data element. A phenotype data element usually does not require such level of sophistication but spans multiple concept domains.

For example, there is no straightforward way of representing the phenotype variable “number of sisters who had breast cancer” with the SNOMED-CT concept models. The most relevant model is the *Situation with Explicit Context* model, as this variable is to capture family history information. However, the *Situation with Explicit Context* model covers only a part of the variable, “sisters had breast cancer.” Full representation would require combining multiple SNOMED-CT concept models or modifying them by introducing additional attributes, increasing the complexity of modeling while decreasing fidelity toward the standards upon which the model is built.

**Figure 3:**
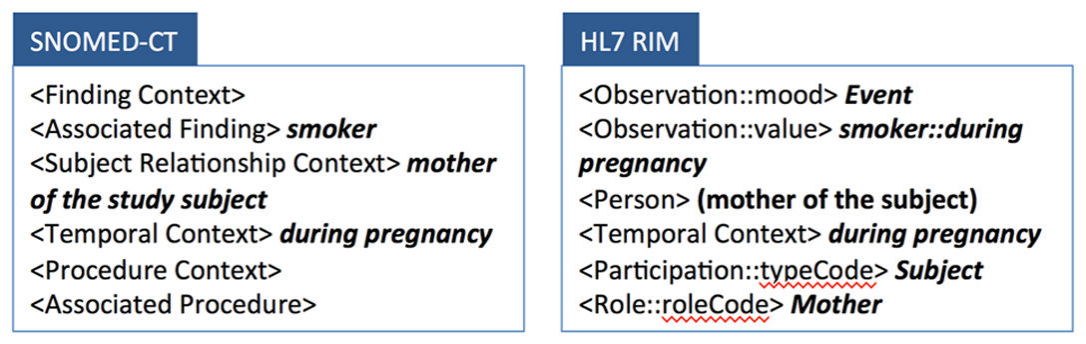
Representing “mother smoked when she was pregnant” using SNOMED-CT and HL7 RIM. SNOMED-CT concept models provides a high level of sophistication for representing a unit of clinical concept, but it lacks a way of representing a phenotype data element, which usually does not require the level of sophistication but spans to multiple concept domains. Here we showed that the SNOMED-CT concept models do not include an explicit *subject* *of* *information* attribute thus subject of the finding is described using the *relationship* *context* attribute. HL7 RIM, an integrate model of healthcare domain, does not provide a complete set of attributes for the concept. HL7 RIM stores the key concepts “smoking during pregnancy” in an unspecific attribute “value” without specifying semantic roles of each concept.

On the other hand, RIM is an integrated model that encompasses the entire healthcare domain. However, it does not provide a complete set of attributes for the concepts represented by its classes. In RIM, many conceptual attributes are represented through the terminology systems used for encoding the values of its class attributes [23,24,25,26,27,28]. Figure **3** shows an example of modeling a phenotype variable “mother smoked when she was pregnant” using a SNOMED-CT concept model and HL7 RIM. The SNOMED-CT concept models do not include an explicit *subject of information* attribute thus subject of the finding is described using the *relationship context* attribute. While the HL7 RIM explicitly represents the patient’s mother using the Person and Role classes, it stores the key concepts “smoking during pregnancy” in the “value” attribute of the Observation class without specifying its semantic role.

**Figure 4:**
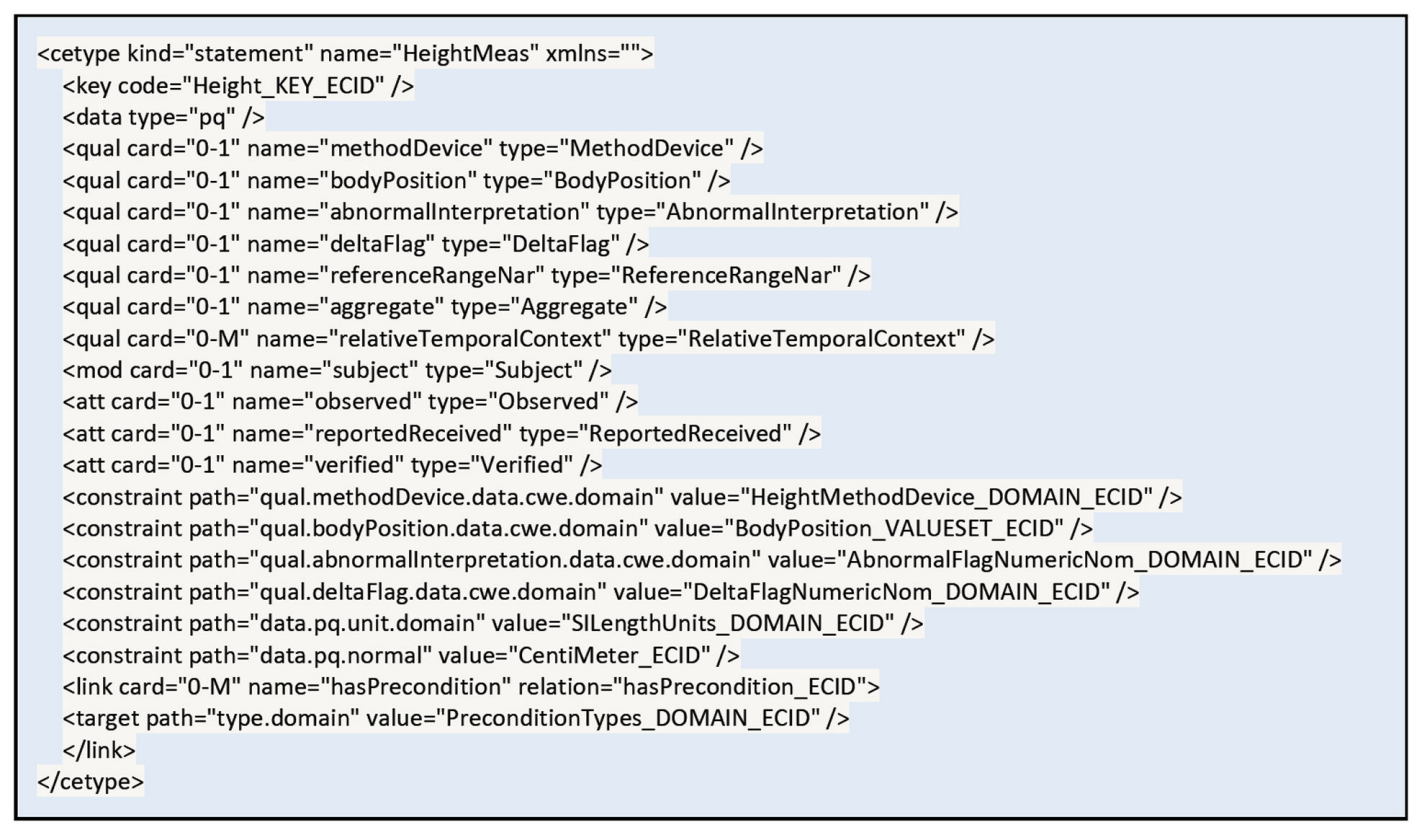
Structure of the HeightMeasure CEM. (**http://intermountainhealthcareorg/CEM/Pages/Detail.aspx?NCID=520862031&k=height**.) CEM represents height measurement with sufficient details through various attributes and qualifiers/modifiers.

Clinical Element Models (CEM) provides a logical structure for representing clinical data. CEM serves as the basis for retaining computable meaning during data exchange between different systems, and was originally designed to support sharing computable meaning when clinical data are applied to decision support [4]. CEM consists of abstract instance models that represent instances of medical data at a general level, and abstract constraint models that further specify the general medical instances with a set of constraints. The current CEM is designed to provide a means of capturing the computable meaning of clinical information in an electronic medical record (EMRs) system in a consistent and robust manner [29]. It provides flexible and comprehensive ways to represent wide ranges of clinical data with sufficient detail, using various attributes and qualifiers/modifiers. Figure **4** illustrates that *height* can be specified in detail through the use of various qualifiers and attributes in CEM [29].

**Figure 5:**
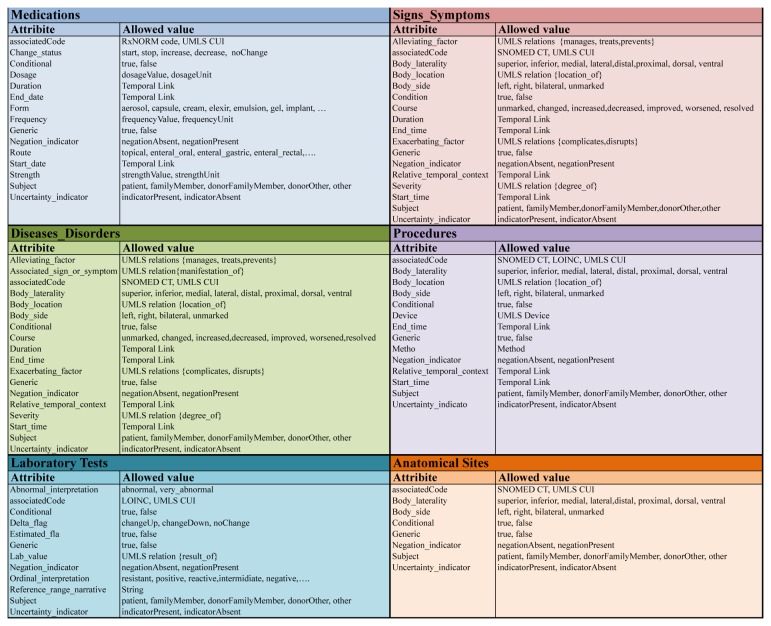
Six CEM template models. Template models that serve as the basis for creating a CEM are available in six domains: *Disease and Disorders*, *Procedures, Signs and Symptoms, Medications, Anatomical* *Sites*, and *Laboratory* *Tests*. Detailed attributes and qualifiers/modifiers in these models are shown.

Template models that serve as the basis for creating a CEM are available in six domains: *Disease and Disorders*, *Procedures, Signs and Symptoms, Medications, Anatomical Sites*, and *Laboratory Tests* (Figure **5**) [30]. The computationally friendly nature of the CEM approach has been recognized as a useful feature for standardizing EHR data and successfully adopted as a type system for NLP processing in SHARPn [14,15,16]. However, its applicability to the phenotype variables generated from research has not been tested. The goal of this study was to test the feasibility of using CEM as a type system for NLP algorithms that process phenotype variable descriptions in dbGaP.

## Methods

This study was conducted in two phases. In Phase I, we tested the feasibility of representing phenotype variables in dbGaP with CEMs. For the first phase of the feasibility testing, we used phenotype names that the dbGaP team abstracted from the phenotype variables submitted to them [31]. These phenotype variables were manually generated thus less idiosyncratic yet more comprehensible than the original dbGaP variables. A few examples of these phenotype names are presented in Table **2**. We will refer to this set of phenotype a “phenotype pilot set” from this point forward. We used the phenotype pilot set as a training set for reviewers. We also used it for initial assessment of the scope of CEM to evaluate whether CEMs could cover phenotype information in our study. In Phase II, we modeled 200 original phenotype variables selected from two phenotype data dictionaries in dbGaP using the CEM template models.

**Table 2:** Examples of phenotype names in the phenotype pilot set and phenotype variable descriptions in dbGaP.

**Phenotype pilot set**	**dbGaP**
Waist/hip ratio in Type II Diabetes Mellitus Cases	Total prednisone bursts since last visit
Human episodic memory	Child had atopic dermatitis for 2 yrs and was seen by a doctor for it
Immunoglobulin A nephropathy	No. of positive core skin tests (all tests) at Follow-up
Hip geometry, neck section modulus, gender differentiated in females	Treatment group assigned at Baseline was LABA arm

### A. Phase I: representing the phenotype pilot set to existing CEM

#### 1) Mapping phenotype names to existing CEM

We retrieved 379 unique phenotype names from the phenotype pilot set and mapped them to the existing CEM. Three reviewers (KL, MT, MR) trained in biomedical informatics conducted mapping. First, the three reviewers were trained in mapping using 50 randomly selected phenotype names. The results from mapping this training set were collaboratively reviewed with two additional reviewers (HK, MC) and disagreements were resolved. After reaching complete agreement in mapping another 15 phenotype names, the three reviewers split the remaining phenotype names and independently mapped them to CEMs.

The reviewers were instructed to select the closest matches when exact matches were not found. The reviewers then specified levels of matches with one of the followings: *exact match, broad match*, and *narrow match*, which are the categories widely adopted in studies evaluating content coverage of standardized terminologies [8,32,33].

*Exact matches* indicate that the selected CEM has exactly the same meaning as the mapped phenotype variable. For example, the phenotype name “systolic blood pressure” mapped to the *SystolicbloodPressureMeas* CEM in an *exact match*. *Broad matches* indicate that the selected CEM had a more general meaning than the mapped phenotype variable. For example, the phenotype name “myocardial infarction” mapped to the *HealthIssue* CEM in a *broad match*. *Narrow matches* indicate that the selected CEM had a more specific meaning than the mapped phenotype variable. The phenotype name “myeloperoxidase” mapped to the *CellsMyeloperoxidase100CellsNFrPtXXXQnLabOb* CEM is an example of the *narrow matches*.

In addition to selecting the closest matches and specifying the levels of matches, we also investigated why *broad matches* and *narrow matches* arose. Unlike content coverage evaluation of a terminology system, which deals with semantic coverage of a single concept, the phenotype names we dealt with in this study consisted with multiple concepts. For each phenotype name, we first identified theme and modifier of phenotype name and of its mapped CEM, then determined whether and at what level the *broad match* or *narrow match* is caused by theme or modifier. The levels were recorded as *broad, exact, narrow, missing modifier, or not applicable*. For example, while phenotype variable name “myocardial infarction” mapped to CEM *HealthIssue*, it was deemed a *broad match* because the theme “health issue” is more general concept than “myocardial infarction”. Modifiers did not affect this matching level, as this case does not have one.

Phenotype name “Mean corpuscular hemoglobin concentration (MCHC)”, which mapped to *ErythrocyteMeanCorpuscularHemoglobinConcentrationMCncPtRBCQnAutomatedCountLabObs CEM* is an example of *narrow match*. In this case, the theme of phenotype name (i.e., “Mean corpuscular hemoglobin concentration (MCHC)”) and the theme of mapped CEM (i.e., “*MeanCorpuscularHemoglobinConcentration*”) *are identical but*, the mapped CEM has an additional modifier “*MCncPtRBCQnAutomatedCountLabObs*” *making the CEM have more specific meaning*. Therefore, this match is deemed *narrow* match.

#### 2) Representing phenotype names using a CEM template model

From the pilot set, we selected 50 phenotype names that were not mapped to an existing CEM and classified them into one of six categories that represent the six CEM template models shown in Figure **5**. Three reviewers (KL, MT, HK), who also had participated in Phase I, were trained with the CEM template models by modeling these 50 phenotype names using a relevant CEM template model.

### B. Phase II: representing phenotype variable descriptions in dbGaP using CEM template models

In this second phase, we investigated whether CEM template models could be applied to formalize the dbGaP variables. We retrieved 200 non-demographic phenotype variable names and descriptions from two data dictionaries of one pulmonary study registered to dbGaP. Two reviewers (KL, MT), who had participated in the modeling exercise in Phase I, conducted the modeling of the 200 phenotype variable descriptions (100 each) using a relevant CEM template model (Figure **3**). Another reviewer (HK), who had also participated in Phase I, reviewed and verified the accuracy of the modeling of the 200 variable descriptions.

## Results

### A. Representing the phenotype name pilot set to CEM

More than half (63%) of the 379 “phenotype names” from the phenotype pilot set were mapped to CEMs. However, the majority (60%) of these matches were broader matches (i.e., mapping to a more general CEM) [34]. The detailed mapping results are presented by phenotype categories in Table 3. Almost all disease variables were mapped to the *HealthIssue* CEM as *broad matches*, since no disease-specific CEM satisfying our needs was available. For analysis of *broad matches*, our results showed that 133 out of 143 (93%) were deemed broad due to *broad* themes, among which 117 (88%) were the *Diseases and Disorders* related variables mapped to *HealthIssue* CEM. Many of the *Laboratory Test* phenotypes were mapped to multiple more-specific CEMs (i.e., narrow matches) because *Laboratory Tests* CEMs carry very detailed test related information based on Logical Observations, Identifiers, and Codes (LOINC) [35]. For example, there are a number of CEMs on glucose level tests, which are specified with specific time points of test (e.g., 2 hours post prandial, 4 hours post prandial). The phenotype name “glucose level test” was not specified with temporal information in the phenotype pilot set. Among 47 narrow matches, 44 (93.6%) were deemed *narrow* due to the additional modifiers, and majority of them (97.7%) were *Laboratory Tests* related variables.

**Table 3:** Results of mapping phenotype names to CEM.

**Phenotype categories**	**Exact**	**Broad**	**Narrow**	**Related**	**Not mapped**	**Total**
Diseases and Disorders	0	116	0	5	7	128
Procedures	0	0	0	0	0	0
Signs and Symptoms	2	19	2	2	56	81
Medications	0	0	0	0	0	0
Anatomical Sites	0	0	0	0	0	0
Labs	20	2	44	10	21	97
Other Findings	4	6	1	7	32	50
Unknown	0	0	0	0	23	23
**Total number**	26	143	47	24	139	379

There were 24 non-exact matches that did not fit to either *broad* or *narrow matches*. For example, the phenotype name “Viscosity” was mapped to the *SerumViscosityViscPtBldQnLabObs* CEM. Because this “phenotype name” did not provide sufficient information on the specimen type, the reviewers were unable to determine the level of match for this mapping. Therefore, we introduced a new category of “related match” to capture this type of matches.

Fifty phenotype names that did not belong to any of the six categories were grouped into the *Other Findings* category. A few examples of phenotype names are “age at death,” “HIV-1 time to progression,” “HLA-C gene expression,” “biologic age by osseographic scoring system,” and “recombination rate, gender specific in males.” Eighteen out of fifty phenotype names in the *Other Findings* category were mapped to CEMs.

There were 139 phenotype variables (37%) that did not map to CEM. Fifty-six out of 139 (40%) were in the category *Signs and Symptoms*. A few examples of unmapped *Signs and Symptoms* phenotype names include “human episodic memory” and “Cognitive performance, Boston Naming test.” There were 21 out of 139 (15%) unmapped phenotype names belonging to the *Laboratory Tests* category. For example, phenotype names such as “plasma CD40 ligand” and “platelet aggregation (collagen induced)” were not mapped to any CEMs.

Twenty-three phenotype names were not mapped to any CEM due to the lack of sufficient information (“*Unknown*”). “mean ratio” and “polytomous analysis” are a few examples of such cases. In summary, two-thirds of the phenotype names in our pilot set were mapped to CEMs with a small fraction of exact matches (6.9%).

### B: Representing the phenotype variable descriptions with CEM template models

We conducted the modeling of the 200 non-demographic phenotype variables selected from a pulmonary study in dbGaP (Table **4**). When categorized by topic, 59% (N = 118) of the 200 variables fell into the non-disease/disorder related *Findings*. A small number of variables were classified as *Medications*-related (N=4, 2%) or *Laboratory tests* (N=16, 8%) variables. About 26% of the variables (N=52) were deemed irrelevant to this study on modeling, as they represented study-specific information (e.g., visit number used for baseline visit, participant assigned to combination therapy group) or workflow related information specifically for study follow-ups (e.g., number of days since last visit, total ER visits). These variables were deemed irrelevant to the representational responsibilities of CEM, and were thus excluded.

**Table 4:** Categories of the phenotype variable and relevant CEM template models used.

**Topics**	**Number of variables**	**Percentage (%**)	**CEM template models used**
Diseases and Disorders	2	1	Diseases and Disorders
Findings (excluding Disease or Disorder)	118	59	Signs and Symptoms
Medications	4	2	Medication, Signs and Symptoms
Laboratory tests	16	8	Laboratory Tests, Signs and Symptoms
Not applicable	52	26	−
Unknown	8	4	−
**Total number**	200	100	−

We were unable to classify or model 8 variables (4%), as their descriptions did not provide sufficient Information. A few examples of such cases are phenotype variable “affection status” and “affection status in PEAK.”

In summary, except for 60 (30%) phenotype variables that were either irrelevant to CEM modeling or unclear in their meaning, all phenotype variables (N=140, 70%) were represented with CEM template models.

We found that none of the phenotype variable descriptions from the 200 variables selected in these studies were mapped to *Procedure* CEM template model. To test the feasibility of representing procedure-related variables using the *Procedure* template model, we manually selected an additional 15 procedure-related phenotype variable descriptions and modeled them. A few examples in this category are “Surgery to remove one ovary after natural menopause,” “Have surgery for snoring surgery,” and “Child jaw surgery.” All 15 phenotype variable descriptions were able to be mapped to *Procedure* CEM template model. The most commonly-used attributes were *body location, device, method, relative temporal context* and *subject*.

## Discussion

Although direct mapping of phenotype names to an existing CEM yielded a very small number of *exact matches*, modeling with the CEM template models covered a majority of the phenotype variable descriptions we tested.

**Figure 6:**
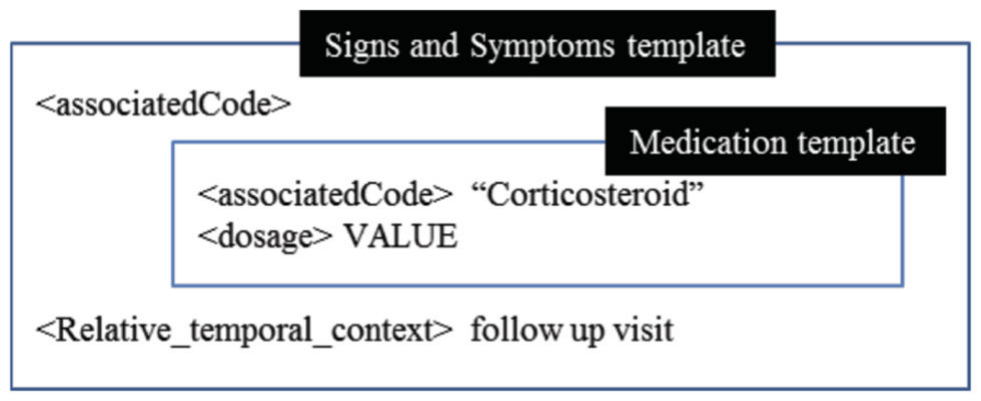
Nested modeling of "Corticosteroid dose at follow up." We modeled a phenotype variable description “Corticosteroid at follow up visit” using integrated multiple relevant CEM template models, including *Signs and Symptoms* CEM template model and *Medication* template model.

During the modeling process, however, we took note of several challenges. First, there was a slight difference between representing phenotype data as clinical data (i.e., as in CEMs) and representing it as research data (i.e., as in dbGaP). The former was often aggregated and reformatted into the latter, to meet data analysis and workflow management demands in research. We expect that many such cases can be resolved by modeling with multiple template models, which can be integrated in a nested fashion, as illustrated in Figure 6.

We are aware that combining multiple CEM template models as described above is not the best way of using the CEM template models. However, we attempted various approaches to utilizing CEM template models because our final goal was not to create new CEM, but rather to develop an NLP-type system that algorithmically standardizes the phenotype variables in dbGaP.

Second, most of the phenotype variables have the nuance of “*Findings*” regardless of their main topic. For example, although a phenotype variable “number of prednisone bursts since last visit” has medication-related information as a main topic, its true intention is to capture the total number of administered prednisone bursts between visits. We first modeled this using the medication template by treating “since last visit (until present)” as duration and the total number of administrations as frequency. However, we also modeled this variable as a “*Finding*” using the *Signs and Symptoms* template, considering that this represents information aggregated from individual prednisone burst administration instances.

This discrepancy stems from the fundamental difference between the clinical data items for which CEM are designed to model, and the phenotype variables collected through GWAS: the former are usually created from discrete instances of clinical events or observations as they occur. However, the latter are the data items processed for research use, meaning that they are generated post-hoc of events or observations often by aggregating or interpreting raw data (i.e., discrete instances of events or observations).

Similarly, a phenotype variable “Log_10_ blood eosinophils at Follow-up” was modeled in two ways: using the *Laboratory Tests* template to capture the topic and the *Signs and Symptoms* template to represent its nuances as a “finding.” The temporal information “at follow-up” was presented with the *Signs and Symptoms* template using *relative temporal context* attribute. Specific constraints for value representation such as *Log*_*10*_ could be accommodated using additional qualifiers within the model. However, we think that metadata on value constraints is a better means to capture this information, as it is not a semantically essential component of the variable.

A similar challenge to applying CEMs to represent clinical data for research purposes was noted in the SHARPn project [15]. The SHARPn researchers suggested additional or different data requirements for particular secondary data use cases, since CEMs were originally created to retrieve EHR data [15,16]. The SHARPn team has been involved in revising or extending CEMs to meet the secondary data uses and has noted that creating common models to normalization of data is much needed but a big challenge [16,36].

Finally, we realized that many attributes of the CEM template models were not utilized in modeling the phenotype variables from dbGaP. The CEM template models are designed to express clinical data with a sufficient level of detail and thus provide a rich set of attributes that can be used to specify clinical events and observations. Despite CEM being less sophisticated than terminology models like SNOMED concept models, their expressivity still made the modeling exercise unnecessarily complex.

On the other hand, these models lacked an attribute dedicated for the main topic concept of a phenotype variable. With CEM templates, topic concepts are modeled with “associated code” attribute, which is to contain not only the main theme of the data element but also the entire data element in a pre-coordinated concept using a standardized concept code. For example, we modeled the finding variable “age of mother first diagnosed with breast cancer” using Signs and Symptoms model, as it is the most relevant to representing findings variables. The main topic “age” was modeled with “associated code,” “mother (of the patient)” was modeled using the subject attribute, and “first diagnosed with breast cancer” was modeled using the “relative temporal context” attribute. However, putting the entire variable “age of mother first diagnosed with breast cancer” with the “associated code” attribute is another legitimate way of modeling.

We have to note here that there was a CEM update in March of 2013, after we completed this study. In order to determine whether our findings still hold with the revised CEMs, we selected 110 from the 379 variable names that we used for the phase I of this study and mapped them to the revised CEMs.

We did not find significant differences in the mapping except that revised CEMs provided more *exact matches*, since specific disease-related CEMs such as *CoronaryHeartDiseasseAssert* and *DiabetesMellitusTypeOneAsser* were added to the revised version. However, most disease-related variable names were still mapped to *HealthIssue* as *broad matches*. We also found a few more non-disease related *exact matches* for the previously unmapped phenotype variables because the revised CEMs contained additional items such as *ExerciseStressTestResultAssertextends, ObservationAssert, FIMScoreLocomotionWalkingWheelchairMeas*, and *FIMScoreMemoryMeas*, This demonstrated that our original findings of the direct mapping between CEMs and the dbGaP phenotype names are still relevant.

The modeling process reported in this study was done manually and served as a first step in testing the feasibility of using an existing information model for clinical data like CEM to standardize phenotype variables based on their free text descriptions. Our ultimate goal was to algorithmically formalize variable descriptions into an information model, in this case CEM template models, to support further NLP processing.

Although conducted on a small scale with 215 phenotype variables, this exercise provided valuable insight into the use of CEM for formalizing phenotype variables. Based on the outcome of this exercise, we decided that developing our own information models for phenotype variables by benchmarking existing standard models would provide more meaningful results. By manually annotating a large number of phenotype variables using the attributes defined in SNOMED and CEM, we have developed custom information models for demographic variables and findings variables with only relevant attributes.

**Figure 7:**
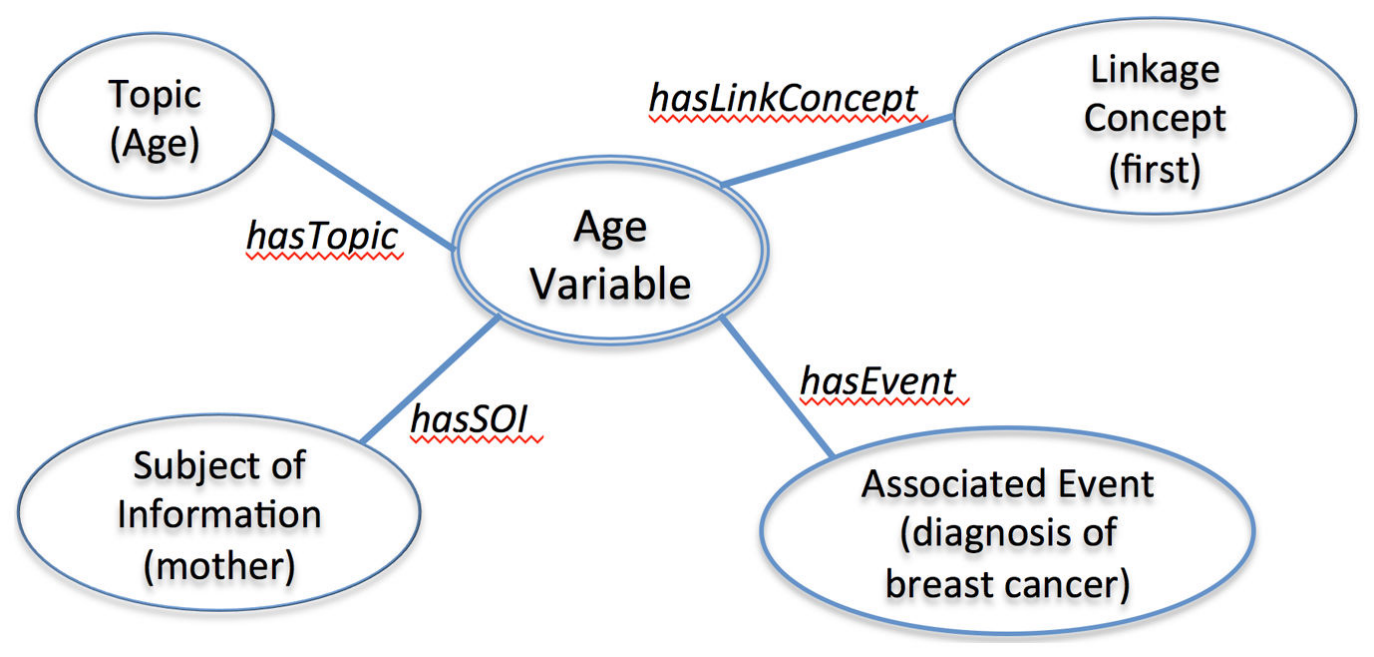
In-house developed information model for Age related finding variables. The core attributes of in-house developed information model include Topic, Subject of Information, Associated Event, and Linkage Concept. A phenotype variable example “age of mother first diagnosed with breast cancer” is represented with the model. As shown, “age” is identified as Topic, “mother” is identified as Subject of Information, “diagnosis of breast cancer” is identified as Associated Event, and first is identified as Linkage Concept.

Figure **7** is our in-house developed information model for age-related findings variables. The previous example of “age of mother first diagnosed with breast cancer” was successfully represented with this model. This model was proven to be successful at representing the key concepts of phenotype variables with a high level of accuracy (92%) [37]. The evaluation of the findings model is currently in progress.

We have identified *key topic* concepts with 70% accuracy and *the subject of information* concepts with greater than 95% accuracy based on UMLS concept mapping and heuristic rules. We are currently focusing on improving the topic concept identification. There are also some existing NLP topic identification tasks that are more challenging than others. For example, Percha et al. used a series of regular expression based rules to classify mammography reports into BI-RADS breast tissue composition categories (e.g. fatty, dense), achieving an accuracy of >99% [38], whereas Harkema et al. achieved an average accuracy of 74% when extracting complex variables relevant to measuring the quality of colonoscopy exams (e.g. “had the patient had a previous colonoscopy?”) [39].

## Conclusions

Reuse of data in dbGaP will facilitate novel scientific discoveries and reduce the cost of research involving integration of genotype and phenotype information. However, nonstandard representations of phenotype variables in dbGaP constitute a major barrier for reusing the data. As a first step towards addressing the issues of unstandardized phenotype variables in dbGaP, we explored the possibility of formalizing phenotype variable descriptions using CEM. Although the use of existing information models of CEM did not fully cover our phenotype variables, it provided a fundamental approach for representing phenotype variable descriptions, based on which we are developing information models for standardizing phenotype variable descriptions.
